# Does richness lose its luster? Effects of extensive practice on semantic richness in visual word recognition

**DOI:** 10.3389/fnhum.2012.00234

**Published:** 2012-08-14

**Authors:** Ian S. Hargreaves, Penny M. Pexman

**Affiliations:** Department of Psychology, University of CalgaryCalgary, AB, Canada

**Keywords:** lexical decision task, practice effects, semantic richness, visual word recognition, reaction time

## Abstract

Previous studies have reported facilitatory effects of *semantic richness* on word recognition (e.g., Yap et al., 2012). These effects suggest that word meaning is an important contributor to lexical decision task (LDT) performance, but what are the effects of repeated LDT practice on these semantic contributions? The current study utilized data from the British Lexicon Project (BLP) in which 78 participants made lexical decision judgments for 28,730 words over 16 h. We used linear mixed effects to detect practice-driven changes in the explanatory variance accounted for by a set of lexical predictors that included numerous indices of relative semantic richness, including imageability, the number of senses and average radius of co-occurrence (ARC). Results showed that practice was associated with decreasing effects of predictors such as word frequency and imageability. In contrast, ARC effects were only slightly diminished with repeated practice, and effects of the number of senses and the age of acquisition were unaffected by practice. We interpret our results within a framework in which variables may dynamically influence lexical processing and the post-lexical decision making mechanisms that also contribute to LDT performance.

## Introduction

Over the past several decades, considerable research attention has been devoted to the study of visual word recognition. As a result, there are now a number of well-established findings in the word recognition literature, and consistent behavioral findings have generally been interpreted as evidence for the stable underlying representational structure of the word recognition system. For instance, consistent behavioral effects of different word characteristics, such as word length effects (faster lexical decisions for shorter words, e.g., New et al., 2006), frequency effects (faster lexical decisions for words that appear more frequently in language, e.g., Balota et al., 2004), and semantic richness effects (faster lexical decisions to words associated with more semantic information, e.g., Pexman et al., 2008) have fuelled assumptions about contributions made by these kinds of information to the process of recognizing words. Indeed, these are among the standard word recognition effects that all models of word recognition are designed to explain.

What is potentially more challenging for models to accommodate, however, is the possibility that there is variability in the process as a function of context or experience, and it is this variability that is the focus of the present work. There is evidence that visual word recognition is a dynamic process; participants can make adjustments to this process in order to optimize performance under various task conditions. For instance, in the standard version of the lexical decision task (LDT), the non-word stimuli are orthographically similar to words (word-like letter combinations, e.g., SLINT) but do not share the sound of a real word if pronounced. If the non-words are made more similar to words, for example by using pseudohomophones (non-words that do sound like real words if pronounced, e.g., BRANE), then several changes in LDT performance can be observed: latencies are slower for both word and non-word responses, and certain behavioral effects (e.g., the word frequency effect) are reliably larger (e.g., Stone and Van Orden, 1993; Lupker and Pexman, 2010).

Similarly, the structural overlap between non-words and words has been shown to create a systematic bias in LDT responses. Keuleers and Brysbaert (2011) designed an algorithm capable of successfully predicting the likelihood of generating a “word” or “non-word” response based solely on the structural similarity of the current trial to past trials (whether “word” or “non-word”). They found that the choice of non-words could bias responses; when non-words were generated from real words (e.g., by manually changing one or two letters of real words as in the English Lexicon Project-ELP; Balota et al., 2007) the high degree of similarity between words and non-words led to a counterintuitive bias to respond “word” when presented with a non-word (and vice versa). This bias also predicted behavioral slowing for both “word” and “non-word” responses in the ELP data and could be mitigated by reducing the structural similarity between word and non-words (e.g., by using the Wuggy algorithm to generate non-words; Keuleers and Brysbaert, 2010). This suggests that participants are implicitly tracking systematic trends in the structural properties of items in order to optimize decision-making in the LDT.

Further, Kiefer and Martens (2010) showed that even purportedly unconscious effects in word recognition can be modulated by context. That is, Kiefer and Martens examined masked semantic priming effects in LDT, involving faster latencies and attenuation of the N400 ERP component for related (table-chair) compared to unrelated (car-hen) targets. Context was manipulated by a perceptual induction task that required directing attention to either semantic or perceptual features. Results showed that semantic priming (in both behavior and ERPs) was enhanced following a semantic induction task and was attenuated following a perceptual induction task. These authors interpreted their results as consistent with the attentional sensitization model, by which top–down mechanisms enhance or attenuate different processing streams in order to facilitate processing that is compatible with higher level goals.

There is also evidence that the word recognition process is shaped by a reader's lexical experience. The effects of practice or experience on word recognition have typically been studied between-subjects, by comparing word recognition behavior in individuals who differ on some experience dimension. For instance, Yap et al. (2011) examined the relationship between readers' vocabulary knowledge and their word recognition behavior, using trial-level LDT data from the ELP megastudy (3374 LDT trials for each of 819 participants). Yap et al. reported that readers with higher vocabulary scores were faster to respond, and that higher vocabulary scores were associated with smaller frequency and semantic effects. Once individual differences in processing speed were controlled, however, Yap et al. found that the relationship between vocabulary scores and a composite frequency/semantic measure (comprised of frequency, number of senses, and also semantic neighborhood density) was only marginally significant.

A small number of studies have explored the effects of experience on lexical processing by examining the word recognition performance of Scrabble experts (Halpern and Wai, 2007; Tuffiash et al., 2007; Hargreaves et al., 2012). Scrabble experience provides the opportunity to develop strong word recognition skills. Since, the ability to detect phony plays (when an opponent plays a non-word) in Scrabble is essential to competitive success, Scrabble players develop extensive knowledge of the lexical status of different letter strings. Hargreaves et al. (2012) showed that this knowledge was associated with faster responses and smaller concreteness effects in LDT, and interpreted this to mean that Scrabble experts showed less reliance on the meanings of words in order to judge lexicality. The behavior of these visual word recognition experts highlights the experience-driven nature of visual word recognition. However, although expert and novice groups were matched on a number of related variables (e.g., vocabulary size, exposure to print, and education), the between-subjects nature of this work means that there is always the possibility that the group differences in word recognition behavior are not due to Scrabble experience but rather to some other uncontrolled group difference.

A within-subjects approach to the study of practice effects in lexical processing was adopted in a recent megastudy. Keuleers et al. (2010) examined the effects of practice on word recognition within subjects by comparing performance across 57 blocks of a LDT using 14,089 Dutch words. The authors found that over time, effects of word frequency diminished with repeated practice in the LDT. Interestingly, the influence of practice on effects of word length and the mean Levenshtein distance to the nearest 20 orthographic neighbors was less clear, as neither formed any linear relationship with repeated LDT practice. That repeated practice selectively influenced some, but not other, lexical variables suggests that practice in the LDT may influence the decision on many levels. Indeed, in another megastudy Dutilh et al. (2009) identified numerous influences of practice on decision-making in the LDT. Dutilh et al. examined the effects of practice on word recognition within subjects by comparing performance across the 25 blocks in a 10,000 trial lexical decision study. They reported that practice was associated with faster and less variable response latencies. Further, diffusion model analyses (e.g., Ratcliff et al., 2006; Ratcliff and McKoon, 2008) showed an increased rate of information processing (increased drift rate), decrease in response caution (or narrowing of decision boundaries), and decrease in time required for common processes executed irrespective of the decision (decreased non-decision time) with practice. Non-decision time facilitation was attributed to increased familiarity with the task demands. These results suggest that with extensive practice participants modify lexical decision making processes in order to optimize performance.

### The present study

In the present work we also adopted a within-subjects approach to the study of practice effects in LDT, capitalizing on a recent LDT megastudy of 28,730 trials known as the British Lexicon Project (BLP; Keuleers et al., 2011). Each participant in the BLP made lexical decisions to 14,365 words (and the same number of non-words) over 16 h of testing. As such, the BLP provides the largest dataset currently available with which to examine effects of practice. We anticipated that, as in the Keuleers et al. (2010) and Dutilh et al. (2009) studies, participants would become much faster across response blocks. Indeed, as Keuleers et al. (2011) noted, the practice effect for word trials in the BLP study was around 100 ms. Our particular interest was in whether participants would show changes in their weighting or reliance on different types of lexical and semantic information as they became more and more practiced at making lexical decisions.

To assess this question, we examined participant behavior across blocks of trials (each block included 500 trials) in the BLP. We assessed the extent to which behavior across blocks could be predicted by orthographic variables, including length and orthographic neighborhood characteristics, word frequency (Brysbaert and New, 2009), and semantic richness. The semantic richness dimension we examined in our analysis of the entire dataset was ARC (average radius of co-occurrence). This measure of semantic neighborhood density was developed by Shaoul and Westbury (2006, 2010) and was derived from the HAL model of lexical co-occurrence (Burgess, 1988; Burgess and Lund, 2000). ARC is based on the average distance of a target word to its neighbors (within a threshold) in high-dimensional semantic space. Shaoul and Westbury (2010) reported that LDT latencies were related to ARC values, such that latencies were faster for words with denser semantic neighborhoods.

## Materials and methods

### Dependent measure

Lexical decision data were obtained from the BLP (http://crr.ugent.be/blp/), an online database containing trial-level LDT data for 28,730 monosyllabic and disyllabic words (Keuleers et al., 2011). A full description of the methodology used in collecting the LDT data is available in Keuleers et al. (2011). For the present analysis it is worth noting that BLP participants were instructed to attempt to maintain a consistently high (80%) level of accuracy, a criterion that was made challenging by the inclusion of a large number of low-frequency words (as low as 0.02 per million words). Participants were instructed to try to keep their average RT below 1 s, however, trials did not time out if they exceeded this value. In the subsequent analysis we included only correct responses for words, further, we required these responses to fall between 200 and 1700 ms. Respectively, these criteria excluded 24.3% and <2% of the data.

### Analysis—full BLP dataset

From the original set of 28,730 words, we were able to obtain a complete set of predictors for 25,463 words, and subsequent analysis proceeded with these items. We constructed a linear-mixed effects (LME) model, implemented using the lme4 library (version 0.999375-42; Bates, 2007) in R (version 2.14.2, Bates, 2007; Baayen et al., 2008; R Development Core Team, 2010). Participants and items were treated as random factors. We used an iterative model fitting procedure included in the package *LMERConvenienceFunctions* (Tremblay, 2011) to generate a random effects structure that provided the best combination of goodness of fit and parsimony (i.e., number of parameters), as determined through iterative testing using likelihood ratio tests. In order to characterize the contributions of semantic richness to LDT performance, ARC values (continuous)^1^ were included as a fixed effects factor. We controlled for additional variance by including letter length (continuous), the orthographic Levenshtein distance to the nearest 20 neighbors (OLD20; continuous; Yarkoni et al., 2008), and log transformed frequency in the SUBTLEX corpus (continuous; Brysbaert and New, 2009), as fixed factors. In addition, we controlled for reaction time on the previous trial by including it as a fixed factor (continuous). All predictors were centered to reduce collinearity (Table 1). Finally, to assess the presence of practice effects we included an interaction term between block (continuous) and all other fixed factors. The significance of individual fixed effects parameters was assessed by subtracting the number of fixed effects in the model (12) from the number of observations (773,527). With hundreds of thousands of degrees of freedom the *t* distribution approximates the normal distribution, thus this approach provides a reasonably conservative estimate of statistical significance (Baayen, 2008). Although, block was included as a continuous fixed factor, in order to facilitate the interpretation of any significant interactions between block and another fixed factor we subsequently dichotomized block using a median split. Thus, block was divided into early (less than block 28) and late (greater than block 28 but excluding the shortened block 57) epochs, and a reduced model without the interaction of block was fitted to the data from early and late blocks.

**Table 1:** **Correlations between centered predictor variables and dependent variable for 25,463 words**.

**Variable**	**1**	**2**	**3**	**4**	**5**	**6**
1. Previous trial RT	−					
2. No. of letters	−0.00^**^	−				
3. Orthographic Levenshtein distance (Yarkoni et al., 2008)	−0.00^**^	0.76^***^	−			
4. Log frequency (Brysbaert and New, 2009)	0.00^***^	−0.37^***^	−0.29^***^	−		
5. ARC (Shaoul and Westbury, 2010)	0.00^**^	−0.28^***^	−0.19^***^	0.68^***^	−	
6. LDT RT	0.25^***^	0.11^***^	0.11^***^	−0.25^***^	−0.21^***^	−

*** p <0.001,

** p < 0.01.

## Results—full BLP dataset

The results of the LME modeling are presented in Table 2. We observed longer reaction times when the previous trial's reaction time was longer. In addition, responses were slower when words had greater mean Levenshtein distance to their nearest 20 neighbors. We observed significant facilitation for higher frequency words and words with denser semantic neighborhoods. In addition, there was a main effect of practice, with mean reaction times falling as participants gained more exposure to the LDT.

**Table 2:** **Effect sizes (bs), standard errors (SEs), and t values for linear mixed effects models of lexical decision reaction times to 25,463 words**.

**Fixed effects**	**Overall**			**Early**			**Late**		
	**b**	**SE**	**t**	**b**	**SE**	**t**	**b**	**SE**	**t**
Previous trial RT	0.07	9.23	73.07^*^	0.08	0.00	95.13^*^	0.08	0.00	87.81^*^
Length	−8.58	0.77	−11.14^*^	−6.75	0.75	−8.98^*^	−5.99	0.68	−8.82^*^
OLD20	25.75	2.12	12.18^*^	22.92	2.09	10.95^*^	22.94	1.92	11.96^*^
Frequency	−54.64	1.35	−40.39^*^	−47.84	1.45	−33.08^*^	−42.79	1.37	−31.32^*^
ARC	−119.10	6.98	−17.05^*^	−113.20	6.64	−17.05^*^	−106.80	6.39	−16.70^*^
Block	−1.17	0.16	−7.44^*^						
Block × previous RT	0.0003	0.00	8.47^*^						
Block × length	0.05	0.01	4.25^*^						
Block × OLD20	−0.02	0.03	−0.61						
Block × frequency	0.36	0.02	21.37^*^						
Block × ARC	0.30	0.10	3.04^*^						
**Random effects**	**Variance**			**Variance**			**Variance**
Subject (Intercept)	6615.7			5315.94			5678.35		
Block	1.93			N/A			N/A		
Length	25.28			30.46			24.38		
OLD20	216.37			255.56			213.29		
Frequency	96.71			133.31			120.20		
ARC	2200.60			2397.46			2304.71		
Item (Intercept)	2211.5			2566.56			2128.60		
Block	0.20			N/A			N/A		
Residual	25171.10			27092.25			24607.13		

Note:

* Indicates that the t-value was significant at p < 0.05.

**Figure 1:**
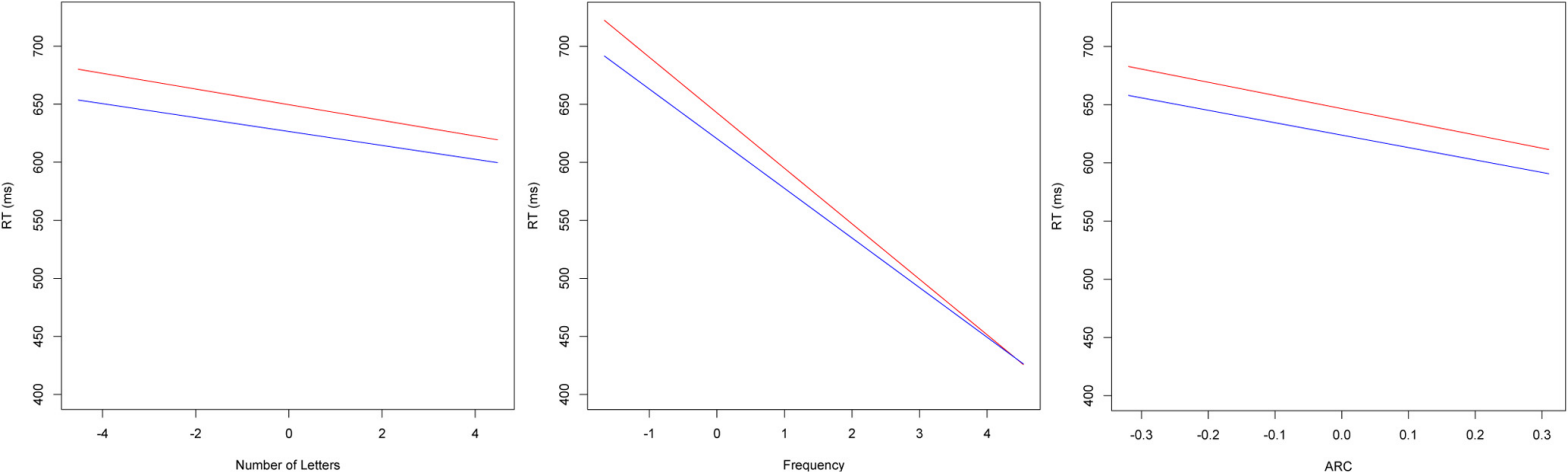
**Plots of fixed effects from a model fitted to LDT reaction times to 25,463 words for early (less than block 28; red) and late (greater than block 28 but excluding the shortened block 57; blue) blocks.** Note: Length, number of letters (centered); Frequency, log SUBTLEX frequency (centered); ARC, semantic neighborhood density (centered).

Importantly, all but one (OLD20) of our main effects were qualified by a significant interaction with block, indicating that the sizes of the lexical and semantic effects were modulated by practice. As displayed in Figure 1, follow-up analyses using a median split of block revealed that the size of length, frequency and semantic neighborhood density (ARC) effects were all reduced in later blocks relative to earlier blocks. These results suggest that participants were reweighting their reliance on different types of lexical and semantic information as they became more and more practiced at making lexical decisions.

While the observation of practice effects is interesting, past investigations of LDT megastudies (e.g., Keuleers et al., 2010) have revealed similar effects of practice on the LDT. Although, the absolute size of this reduction is quite modest, the observation that semantic richness effects are also modulated by practice is a novel finding that raises a question about whether other descriptions of relative semantic richness would show similar practice effects^2^. Numerous studies have revealed that the changes in processing elicited by different tasks can lead to the selective recruitment of different descriptions of semantic richness, with some dimensions contributing to some tasks and not to others (e.g., imageability, the number of senses, ARC; Pexman et al., 2008; Yap et al., 2011, 2012). Similarly, the increased efficiency in the LDT that is purchased with practice may selectively influence some forms of semantic richness but not others. In order to assess this question we performed a separate analysis on a subset of items for which we had a complete set of predictors including several forms of semantic richness: the number of senses a word has (as evidenced by the number of discrete Wordsmyth entries; http://www.wordsmyth.net), a word's rated imageability, and finally a word's semantic neighborhood density as measured by ARC. In addition, we were able to introduce an additional control for the words' estimated age of acquisition.

### Analysis—restricted BLP dataset

From the original set of 28,730 words, we were able to obtain a complete set of predictors for 3723 words, and subsequent analysis proceeded with these items. We constructed a LME model, again implemented using the lme4 library. Participants and items were treated as random factors. We used an iterative model fitting procedure included in the package *LMERConvenienceFunctions* (Tremblay, 2011) to generate a random effects structure that provided the best combination of goodness of fit and parsimony (i.e., number of parameters), as determined through iterative testing using likelihood ratio tests. The number of senses (continuous; Wordsmyth), imageability (continuous; Cortese and Fugett, 2004; Stadthagen-Gonzalez and Davis, 2006), and ARC values (continuous)^3^ were included as fixed semantic richness factors. In order to control for additional variance we included letter length (continuous), the orthographic Levenstein distance to the nearest 20 neighbors (OLD20; continuous; Yarkoni et al., 2008), log transformed frequency in the SUBTLEX corpus (continuous; Brysbaert and New, 2009) and age of acquisition (AoA; continuous; Stadthagen-Gonzalez and Davis, 2006; Cortese and Khanna, 2008) as control variables. Finally, we controlled for reaction time on the previous trial by including it as a fixed factor (continuous). All predictors were centered to reduce collinearity (Table 3). In order to assess the presence of practice effects we included an interaction term between block (continuous) and all other fixed factors. Again, the significance of individual fixed effects parameters was assessed by subtracting the number of fixed effects in the model (18) from the number of observations (129,925; Baayen, 2008). In order to facilitate the interpretation of any significant interactions between block and another fixed factor we subsequently dichotomized block using a median split. Thus, block was divided into early (less than block 28) and late (greater than block 28 but excluding the shortened block 57) epochs, and a reduced model without the interaction of block was fitted to the data from early and late blocks.

**Table 3:** **Correlations between centered predictor variables and dependent variable for 3723 words**.

**Variable**	**1**	**2**	**3**	**4**	**5**	**6**	**7**	**8**	**9**
1. Previous trial RT	−								
2. No. of letters	−0.01	−							
3. Orthographic Levenshtein distance (Yarkoni et al., 2008)	−0.01^*^	0.74^***^	−						
4. Log frequency (Brysbaert and New, 2009)	0.00	−0.25^***^	−0.20^***^	−					
5. Age of acquisition	−0.00	0.22^***^	0.21^***^	−0.69^***^	−				
6. Imageability	0.00	−0.11^***^	−0.12^***^	0.13^***^	−0.48^***^	−			
7. Number of senses	0.00	−0.15^***^	−0.19^***^	0.41^***^	−0.37^***^	0.13^***^	−		
8. ARC (Shaoul and Westbury, 2010)	−0.00	−0.09^***^	−0.07^***^	0.74^***^	−0.48^***^	0.15^***^	0.33^***^	−	
9. LDT RT	0.29^***^	0.02^***^	0.00	−0.10^**^	0.10^***^	−0.05^***^	−0.06^***^	−0.09^**^	−

*** p <0.001,

** p <0.01,

* p <0.05.

## Results—restricted BLP dataset

**Figure 2:**
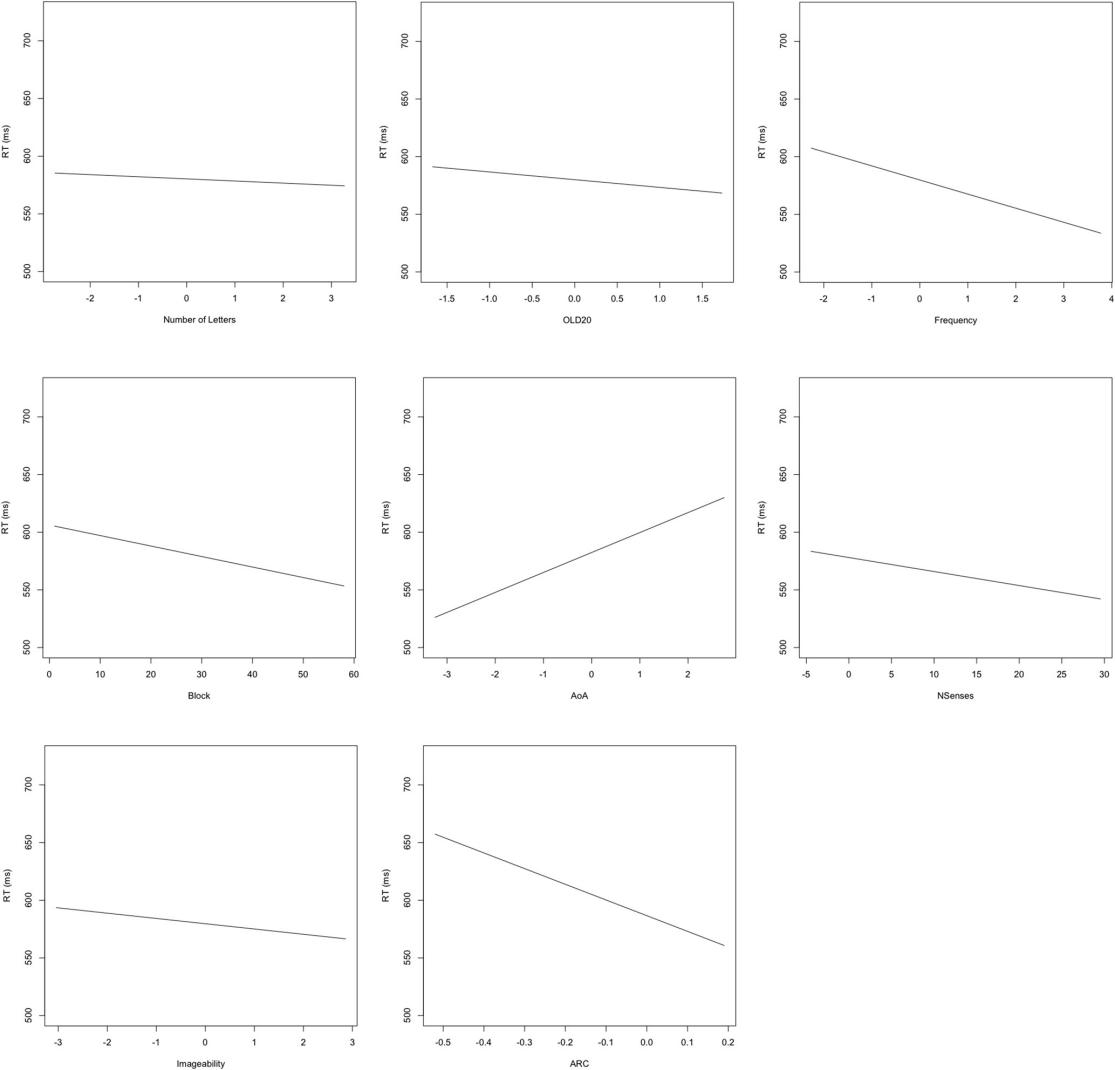
**Plots of fixed effects from a model fitted to LDT reaction times to 3723 words.** Note: Length, number of letters (centered); OLD20, mean levenshtein distance to nearest 20 orthographic neighbors (centered); SUBTLEwf, log SUBTLEX frequency (centered); AoA, Age of acquisition (centered); NSenses, Number of Senses; IMG, Imageability (centered); ARC, semantic neighborhood density (centered).

The results of the LME modeling are presented in Table 4 and in Figure 2. Length was the only predictor that failed to form a significant relationship with reaction time. We observed facilitatory effects of block and frequency as increasing practice and word frequency led to shorter reaction times. Curiously, the effect of OLD20 ran in the opposite direction as that observed with the larger set of items. Words with less dense orthographic neighborhoods showed faster reaction times. In their analysis of the BLP data, Keuleers and colleagues (2010) were unable to find reliable effects of the related construct number of orthographic neighbors. Indeed, our follow-up to the significant interaction suggests that the influence of orthographic neighbors for this set of items may be highly variable, reaching significance in early blocks but not in later blocks. More expected was the observation that words learned later in life (as assessed by AoA ratings) had longer reaction times than those learned earlier in life. All semantic richness variables led to significant facilitation in the expected direction, with more senses, higher imageability ratings and denser semantic neighborhoods all being associated with faster responses in the LDT.

**Table 4:** **Effect sizes (bs), standard errors (SEs), and t values for linear mixed effects models of lexical decision reaction times to 3723 words**.

**Fixed effects**	**Overall**			**Early**			**Late**		
	**b**	**SE**	**t**	**b**	**SE**	**t**	**b**	**SE**	**t**
Previous trial RT	0.06	0.00	26.25^*^	0.08	0.00	40.15^*^	0.09	0.00	41.13^*^
Length	−0.71	1.56	−0.46	−1.34	1.45	−0.92	−2.14	1.19	−1.80
OLD20	−15.25	3.46	−4.41^*^	−9.92	2.92	−3.39^*^	−3.49	2.60	−1.34
Frequency	−17.11	2.29	−7.47^*^	−15.30	2.05	−7.45^*^	−9.36	1.85	−5.07^*^
Imageability	−7.41	0.90	−8.21^*^	−6.24	0.81	−7.71^*^	−2.99	0.69	−4.35^*^
AoA	18.77	1.38	13.63^*^	17.01	1.13	15.04^*^	17.33	1.02	17.08^*^
Number of senses	−1.26	0.24	−5.29^*^	−1.18	0.19	−6.24^*^	−1.28	0.17	−7.64^*^
ARC	−156.60	13.34	−11.74^*^	−142.10	12.51	−11.36^*^	−126.40	11.15	−11.34^*^
Block	−0.89	0.15	−6.08^*^						
Block × previous RT	0.00	0.00	7.91^*^						
Block × length	−0.04	0.04	−1.09						
Block × OLD20	0.30	0.09	3.32^*^						
Block × frequency	0.17	0.05	3.12^*^						
Block × imageability	0.10	0.02	4.51^*^						
Block × AoA	−0.05	0.04	−1.41						
Block × number of Senses	0.00	0.01	0.26						
Block × ARC	0.71	0.28	2.54^*^						
**Random effects**	**Variance**			**Variance**			**Variance**
Subject (Intercept)	4476.37			4495.0			5132.76	
Block	1.62			N/A			N/A	
Length	42.49			59.57			30.59	
Frequency	72.99			90.98			82.52	
Imageability	11.18			16.47			9.18	
ARC	4277.40			5078.81			4135.98	
Item (Intercept)	816.31			870.01			642.62	
Length	20.97			59.14			2.88	
Frequency	331.25			549.31			279.25	
Residual	23358.14			24852.09			22290.12	

* Indicates that the t-value was significant at p < 0.05.

**Figure 3:**
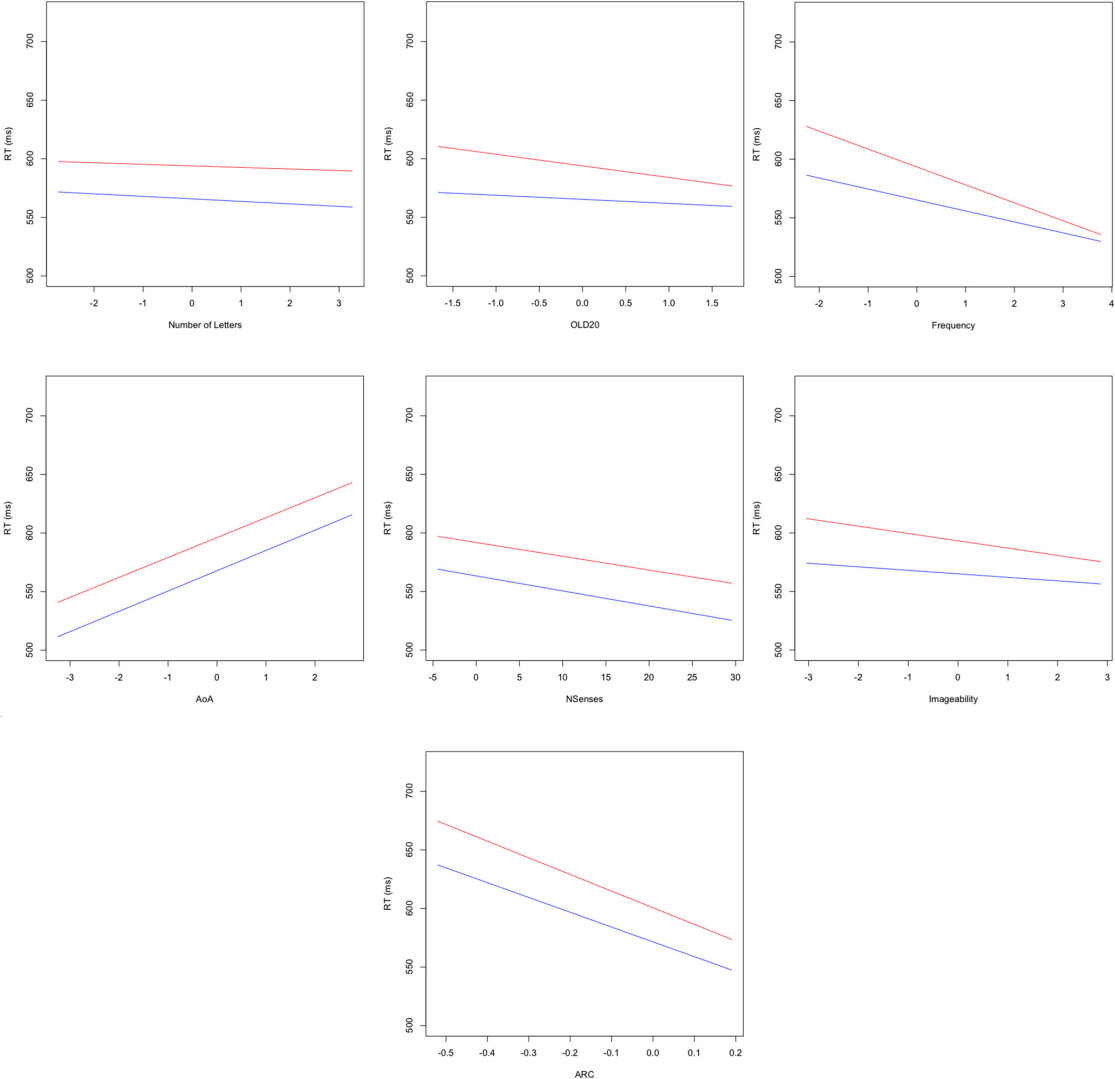
**Plots of fixed effects from a model fitted to LDT reaction times to 3723 words for early (less than block 28; red) and late (greater than block 28 but excluding the shortened block 57; blue) blocks.** Note: Length, number of letters (centered); OLD20, mean levenshtein distance to nearest 20 orthographic neighbors (centered); SUBTLEwf, log SUBTLEX frequency (centered); AoA, Age of acquisition (centered); NSenses, Number of Senses; IMG, Imageability (centered); ARC, semantic neighborhood density (centered).

The main effects of orthographic Levenshtein distance, frequency, imageability and ARC were all qualified by significant interactions with block, indicating that only these variables showed a practice effect. As displayed in Figure 3, follow-up analyses using a median split of block revealed that the sizes of all of these effects were attenuated as participants gained more practice in the LDT. Interestingly, the decreases in the sizes of the effects for orthographic Levenshtein distance, frequency, and imageability with practice were much larger than that observed for the decrease in ARC effects with practice.

## Discussion

The results of the current investigation provide further evidence that extensive practice with the LDT leads to significant facilitation as participants' responses became faster with increasing experience with the LDT. The current results also provide an early look at the influence of practice effects on numerous lexical and semantic dimensions, and indicate that practice-driven optimization of processing in the LDT has a diverse set of consequences. While practice led to clear attenuation of the contributions of some lexical and semantic dimensions (e.g., word frequency and imageability), for other dimensions we observed practice-driven attenuation that, though reaching statistical significance, had limited practical significance (e.g., ARC). Further, for the contributions of other dimensions we observed no appreciable practice-driven attenuation at all (e.g., the number of senses and AoA).

Performance in the LDT is thought to provide a window into the mechanisms that drive lexical processing, but it is also thought to depend upon the contributions of post-lexical decision-making mechanisms (Balota and Chumbley, 1984; Yap et al., 2011). Recent research investigating the influence of repeated practice in the LDT suggests that practice influences both lexical and post-lexical processes (Dutilh et al., 2009). Appealing to the diffusion model, Dutilh and colleagues argued that the influence of practice is observed not only in changes to the speed of information processing (as indexed by drift rate), but also in changes in the overall familiarity with the task demands, which allows participants to dynamically adjust their criteria for what evidence counts as a “word” or “non-word” response. Though interesting, these findings are framed in terms of the specific parameters of the diffusion model, parameters that are not transparently linked to any specific lexical process. The results of the present study add to this literature by qualifying how this practice-driven optimization influences many of the lexical and semantic dimensions that are the focus of contemporary research into visual word recognition.

### Control variables

One present finding of interest is the observation of significant decreases in the contributions of word frequency with practice. We observed a clear facilitatory effect of frequency with increases in word frequency associated with faster RTs. Interestingly, in both the full (25,463 items) and restricted analysis (3723 items) the significant effects of frequency also formed a significant interaction with block, as the size of the frequency effect decreased with repeated practice.

There are numerous potential explanations for this diminishing utilization of frequency information by participants. In diffusion model terms one could argue that faster information accumulation, coupled with narrower decision boundaries, could reduce the amount of time that frequency information has to accumulate, thereby reducing the influence of frequency on LDT RTs. However, one could just as easily argue that the decreasing effect of frequency may indicate that participants were, over time, adjusting to the large number of low-frequency words in the BLP stimulus set, rendering frequency-information less diagnostic of a decision. Both interpretations of the relationship between practice and frequency are admittedly *post hoc*, however, they serve to emphasize that these variables can conceivably influence both lexical and post-lexical processing.

One control variable that did not diminish in its contributions to LDT performance was AoA. Replicating numerous studies (Ellis and Lambon Ralph, 2000), we observed that words rated as being learned earlier in life were associated with faster responses in the LDT. Importantly, the AoA effect was highly consistent, and did not diminish with repeated practice. Though, correlated with word frequency, many researchers have suggested that the effect of AoA reflects a unique contribution that is independent of that of word frequency (Juhasz, 2005; c.f., Zevin and Seidenberg, 2002). Whether AoA effects result from the influence of unmeasured cumulative word frequency (Zevin and Seidenberg, 2002) or have a semantic locus, as suggested by network models of AoA effects (Ellis and Lambon Ralph, 2000; Steyvers and Tenenbaum, 2005), the contributions of AoA are in some cases greater than the contributions of word frequency alone (Juhasz, 2005). The current findings also suggest a clear dissociation between frequency and AoA. As Table 4 shows, there was a 39% reduction in the effect of word frequency between early and late blocks (*b* = −15.30 and −9.36 respectively). In contrast, AoA effects were indifferent to repeated practice, showing no appreciable change between early and late blocks (*b* = 17.01 and 17.33 respectively), despite the overall increase in the speed of participants' LDT responses that accompanied practice. The current findings reveal that an analysis of repeated practice in the LDT might provide important insight into dissociating variables that are highly correlated, and demonstrate that the influence of AoA on LDT response times is pervasive and consistent.

### Semantic richness variables

Replicating findings from several previous studies we observed facilitatory effects for all of our semantic richness variables (Yap et al., 2012). However, while the individual contributions of these variables were consistently facilitatory, each variable responded uniquely to extensive practice in the LDT. In both the full and restricted analyses, ARC emerged as a significant predictor of reaction time in the LDT. This effect was qualified by a significant interaction with practice; however, though ARC effects decreased as participants gained more experience in the LDT the absolute decrease in the size of ARC effects was relatively small. As shown in Tables 2 and 4, between early and late blocks we observed a 6% decrease in the ARC effect for 25,463 items, and a 11% decrease for 3723 items. This stands in contrast to the effect of imageability which, like ARC, reached statistical significance and formed a significant interaction with block. Unlike ARC, the practice-driven decrease in imageability effects was relatively large, with a 52% decrease in the size of imageability effects between early and late blocks. This potential for practice effects to have strikingly disparate consequences for different measures of semantic richness is further highlighted by the finding that facilitatory effects of number of senses do not interact with practice. Indeed, as shown in Table 4, the fixed effects estimates for the number of senses are highly similar between early and late blocks (*b* = −1.17 and −1.28, respectively).

One theme to emerge from studies of semantic representation is that it is often useful to organize semantic dimensions into those that reflect object-based properties (i.e., semantic properties reflecting our immediate sensory experience with real-world exemplars of concepts) and those that reflect language-based properties (i.e., semantic properties reflecting our experiences processing the hierarchical statistical regularities that govern word-to-word usage in natural language; Buchanan et al., 2001). These divisions reflect distinct pathways by which humans can come to acquire and represent knowledge, and a diverse set of evidence suggests that both language- and object-based information can contribute during reading (Paivio, 1971; Buchanan et al., 2001; Solomon and Barsalou, 2004; Pulvermüller, 2010). Interestingly, recent evidence suggests that when task demands favor the shallow processing of meaning (e.g., as in the LDT; Lupker and Pexman, 2010) the language-based system reaches peak activation earlier, and contributes to the decision before the object-based simulation system (Simmons et al., 2008; Louwerse and Connell, 2011). One piece of evidence for this distinction comes from a study by Barsalou and colleagues, who asked participants to list properties of a provided word (Simmons et al., 2008; Santos et al., 2011). They found that the earliest listed associates were linguistically related to the cue, reflecting associative (e.g., *bee* -> *hive*) or phonological relationships (e.g., *self* -> *selfish*). Later associates tended to reflect properties that could emerge from situated simulation, such as properties of the environment (*golf* -> *sunshine*), or physical properties of the objects (*bee* -> *wings*). Subsequent analysis of fMRI data collected while participants were generating associates revealed that early (the first 7.5 s) property generation was moderated by classic language areas (e.g., Broca's area) while later generation (7.5–15 s) involved areas associated with mental imagery and episodic memory. The authors concluded that both language-based and object-based simulation systems contribute to the relatively shallow processing in the property generation task, however, the language-based system makes earlier contributions than the object based-system.

In the LDT, semantic processing is relatively shallow, and is thought to contribute to participants' decisions mostly in terms of feedback to orthography (Pexman et al., 2002). As participants gain experience with the LDT, one expectation is that practice-driven optimization will reduce the relative contributions of feedback, allowing participants to make their decisions while engaging in shallower semantic processing. This expectation is supported by the finding that competitive Scrabble players, who perform the LDT significantly faster than age-matched controls, seem to de-emphasize the role of meaning in their decision as evidenced by a significant reduction in the size of concreteness effects (Hargreaves et al., 2012). If practice driven efficiency leads to shallower semantic processing, those semantic processes that are faster will continue to contribute to the LDT decision (e.g., the language-based processes that reflect our histories of reading words) whereas the contributions of those systems that rely on deeper, situated simulation (e.g., the contributions of imagery information) will be disproportionately disrupted. One interesting feature of the current findings is that the observed dissociations in the effects of practice on the different descriptions of semantic richness also divide themselves between object-based (i.e., imagery) and language-based (i.e., ARC and number of senses) descriptions. The potential for repeated practice to encourage shallower semantic processing may account for the current data, in which practice-driven increases in LDT efficiency are associated with a reduction in participants' reliance on object-based (i.e., imagery) semantic information but this practice does not modulate their reliance on language-based (i.e., ARC and the number of senses) semantic information.

This interpretation, though admittedly *post-hoc*, connects with extant theories of the relative roles of linguistic and embodied information in informing reading, the unique characteristics of LDT expertise found among competitive Scrabble experts, and is also supported by a growing literature that utilizes multiple tasks in order to dissociate the effects of different descriptions of semantic richness (Yap et al., 2012). Numerous studies have highlighted the potential for word meaning to contribute to participants' judgments in the LDT. For years, the finding that words rated as being higher in mental imagery were responded to faster was taken as crucial evidence for the role of imagery-information in lexical processing (Paivio, 1971). The list of semantic dimensions continues to expand. For example, some researchers have taken advantage of surveys of dictionary definitions to characterize variability in word usage (e.g., number of senses; Yap et al., 2012), or have taken advantage of advances in computing in order to derive a variable that characterizes the history of a words' usage in a text-based corpus (e.g., ARC; Shaoul and Westbury, 2010). Like imageability, the contributions of these semantic effects to LDT performance are taken as evidence that these semantic dimensions shape lexical processing. There is mounting evidence that this diverse set of semantic richness variables each account for some unique aspect of meaning, and do not reflect the solitary contribution of a single underlying semantic factor. Utilizing cross-task comparisons, researchers have demonstrated that different descriptions of meaning play a unique role in performance that varies depending upon the demands of the task (Pexman et al., 2008; Yap et al., 2012). The current results add to this effort by revealing that, like task demands, changes in processing that occur with practice-driven optimization in the LDT reveal clear dissociations in the relative contributions made by different characterizations of semantic richness. This observation is supported by a pronounced diversity in the influence of practice on semantic richness effects, with some semantic variables showing large effects of practice (e.g., imageability), others showing more moderate effects (e.g., ARC), and still others showing no effect of practice at all (e.g., number of senses).

Our study provides the first investigation of the effects of repeated practice in the LDT on semantic richness effects. We selected predictor variables based on theoretical importance and the availability of a complete data set in order to maximize the sensitivity of what is an immense within-subjects study. We were able to find a complete set of values for 3723 words, and for a smaller set of predictors, 25,463 out of 28,730 words. This is a substantial improvement over previous investigations of semantic richness, and highlights the power of the BLP dataset. The observation of practice effects has implications for models of visual word recognition, particularly those that utilize only a single dimension of lexicality (e.g., MROM; Grainger and Jacobs, 1996). These models would have a difficult time explaining the present results precisely because we observed a dynamic tradeoff across multiple dimensions of the information utilized by participants in order to maximize efficiency. In order to explain the present findings, models of visual word recognition would need to incorporate the possibility that multiple dimensions of information can be emphasized or de-emphasized as a function of task demands, perhaps in a manner similar to that described in the attentional sensitization model (Kiefer and Martens, 2010).

In summary, the current study reveals that different dimensions of lexical and semantic information can display considerable variability in their utilization by participants over repeated practice. While some dimensions continue to provide information that is consistently diagnostic of a word decision (e.g., the Number of Senses and AoA) other dimensions become less important as the participant gains more familiarity with the demands of the decision, and with the kinds of items in the LDT. This suggests that the contributions of lexical and semantic information towards a lexical decision are dynamic. Though, the current results may be a function of the specific demands created by the LDT, they are consistent with a literature on practice effects that finds the influence of practice to play out across numerous dimensions, even for very basic tasks (Dutilh et al., 2009).

### Conflict of interest statement

The authors declare that the research was conducted in the absence of any commercial or financial relationships that could be construed as a potential conflict of interest.

## Footnotes

^1^http://www.psych.ualberta.ca/~westburylab/downloads/westburylab.arcs.ncounts.html

^2^Our thanks to editor for this suggestion.

^3^http://www.psych.ualberta.ca/~westburylab/downloads/westburylab.arcs.ncounts.html
